# Surgical options for submucosal tumors near the esophagogastric junction: does size or location matter?

**DOI:** 10.1186/s12893-020-00840-6

**Published:** 2020-08-06

**Authors:** Yi-Chun Huang, Chun-Nan Yeh, Ming-Yang Chen, Shang-Yu Wang, Keng-Hao Liu, Chun-Yi Tsai, Ta-Sen Yeh

**Affiliations:** Department of General Surgery, Chang Gung Memorial Hospital, Linkou branch, No.5, Fu-Xing Street, Kweishan District, Taoyuan City, 333 Taiwan

**Keywords:** Submucosal tumor, Esophagogastric junction, Minimally invasive surgery, Gastrointestinal stromal tumor, Gastrointestinal tract

## Abstract

**Background:**

Submucosal tumors (SMTs) of different etiologies exist from esophagus to rectum. Esophagogastric junction (EGJ) is one of the known difficult locations for tumor resection. Although minimally invasive surgery (MIS) is a well-established approach for gastrointestinal surgery, there is no consensus that MIS for resection of SMTs around EGJ is superior to laparotomy. We tried to clarify the factors that determine the surgeons’ choices between these two approaches.

**Methods:**

From January 2002 to June 2016, 909 patients with SMTs underwent resection in our department. Among them, 119 patients (13%) had SMTs around EGJ were enrolled by retrospective review. The clinicopathological features and tumor-related parameters were reviewed and analyzed.

**Results:**

The cohort was stratified into three groups according to the extent of gastrectomy and surgical approaches. The three groups are as following: major gastrectomy (*n* = 13), minor gastrectomy by laparotomy (*n* = 51), and minor gastrectomy with MIS (*n* = 55). The average tumor size was significantly larger in the major gastrectomy group than in the two minor gastrectomy groups; however, there was no difference between the two minor gastrectomy groups (5.33 cm, 4.07 cm, and 3.69 cm, respectively). The minor gastrectomy with MIS required least hospital stay and operation duration also. We re-stratify the two minor gastrectomy groups (*n* = 106) according to the orientation of SMTs around the EGJ into 4 zones. Most of SMTs located on the greater curvature side of the EGJ were resected with MIS (82% versus 18%), whereas SMTs in the other zones were resected more often by laparotomy (59% versus 41%). There was no surgical mortality within the cohort, while minor gastrectomy with MIS yielded least number of leakages among the three groups.

**Conclusions:**

For SMTs around the EGJ, larger tumors (diameter of more than 5 cm) are more likely to be resected with major gastrectomy. To resect SMTs around the EGJ in a wedge-like (minor gastrectomy) fashion, tumors located other than the greater curvature side were more often resected by laparotomy. However, MIS yielded acceptable safety and surgical outcomes compared to conventional laparotomy for SMTs around the EGJ of the same size.

## Background

Submucosal tumors (SMTs) are mesenchymal neoplasms arising from muscular or neural origin [1]. The etiologies of SMTs include gastrointestinal stromal tumor (GIST), leiomyoma, schwannoma, and mucosal malignancy presenting with a submucosal mass, and are difficult to determine by merely endoscopy or computed tomography (CT) [2]. For undetermined SMTs smaller than 2 cm, close follow-up with regular surveillance is suggested [3, 4]. Endoscopic or surgical intervention should be applied for those tumors presenting with interval changes. Minimally invasive surgery (MIS) is a well-established approach to resect gastrointestinal SMTs indicated for surgery [5, 6]; however, there is no consensus for those located around the esophagogastric junctions (EGJ). The resection of tumors near EGJ carries the risk of luminal stricture or leakage [7], which renders MIS restrictive for surgeons. There have been several series focusing on MIS for the resection of SMTs near the EGJ that concluded that it is a safe and rational approach [8–10]. However, in our daily practice, we still observe SMTs in difficult locations that surgeons are reluctant to resect with MIS surgery. We hereby retrospectively reviewed our series and tried to clarify the factors that determine the choice between conventional laparotomy and MIS.

## Methods

### Study population

**Fig. 1:**
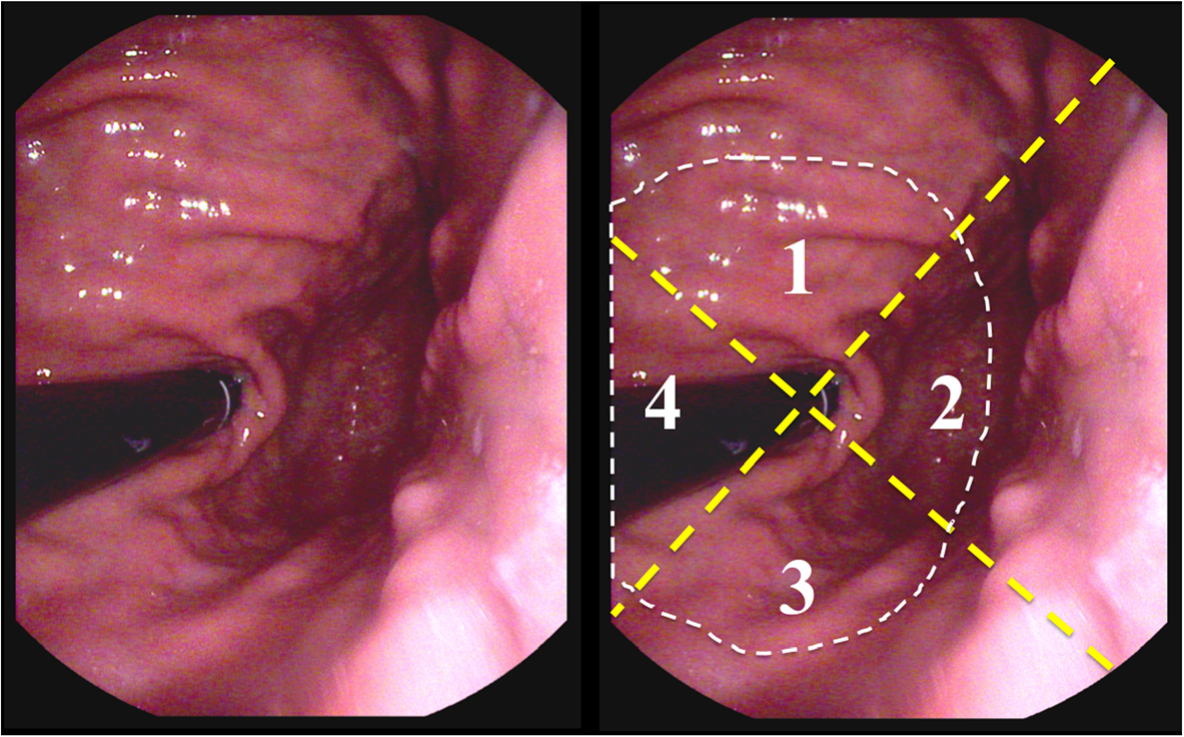
The endoscopic view around the EGJ from the stomach side. The white dotted line indicated the 2 cm distance of gastric side to the EGJ. The two crossed yellow dotted lines illustrated the division of zone 1 to zone 4

By reviewing the registered database of SMTs underwent resection from January 2002 to June 2016 at our department, the Department of General Surgery, Chang Gung Memorial Hospital, Linkou branch, 909 patients with SMTs in the stomach were selected. All these patients received esophagogastroduodenoscopy (EGD) before resection, either in our institute or at local clinics. The images of EGD and the operation records of these 909 patients were reviewed by two surgeons (CY and MY). CT of abdomen, if available, was also reviewed to determine the tumor extent from the seorsa side. The location of EGJ was defined by the endoscopic view from the gastric side [11]. We focused on the tumors located at the gastric side within 2 cm to the EGJ (Fig. 1). There were 119 patients (13%) meet with the inclusion criteria. The indications for resection were symptomatic tumors, biopsy-proven GISTs, or longest tumor diameter larger than 2 cm. The patients’ characteristics, final pathology of the tumor, size and location of the tumor, method of resection, duration of the operation, length of postoperative hospital stay, and morbidity or mortality after the operation were evaluated. The size of the tumor was defined by the longest diameter of the tumor, which was recorded for all patients except for one patient with an extensively necrotic tumor for which the size could not be determined. The last date of follow-up was designated as 31 March 2018. The dataset used/ analyzed in the current study is available from the corresponding author by reasonable request.

### Statistical analysis

Continuous variables are expressed as the mean ± standard deviation and were compared using Student’s t-test. Categorical variables were compared using the chi-square test or Fisher’s exact test, as appropriate. All analyses were performed with SPSS version 21 (IBM SPSS Statistics for Windows, Version 21.0, Armonk, NY: IBM Corp.). *P*-values < 0.05 were considered significant.

## Results

**Fig. 2:**
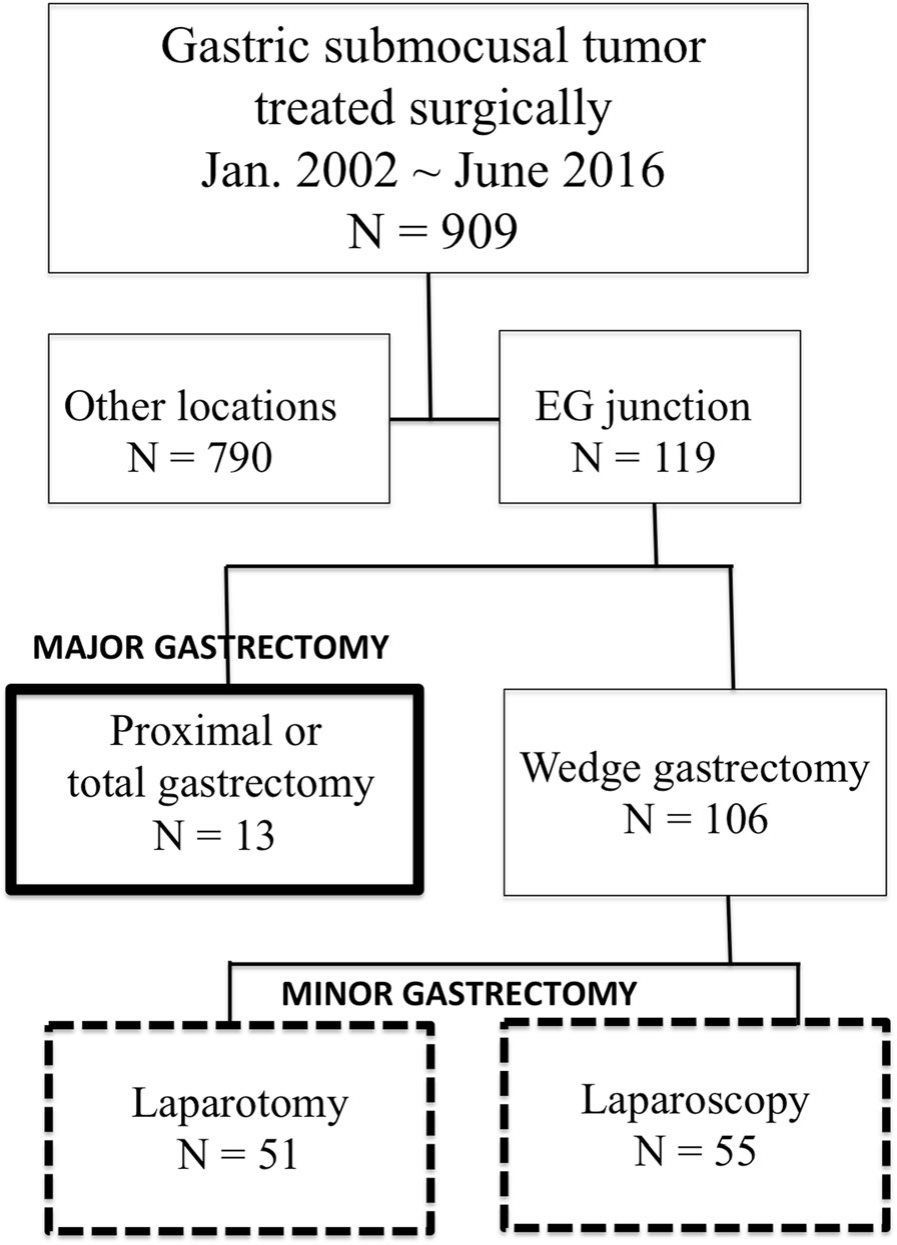
The algorithm of patient selection and stratification

**Fig. 3:**
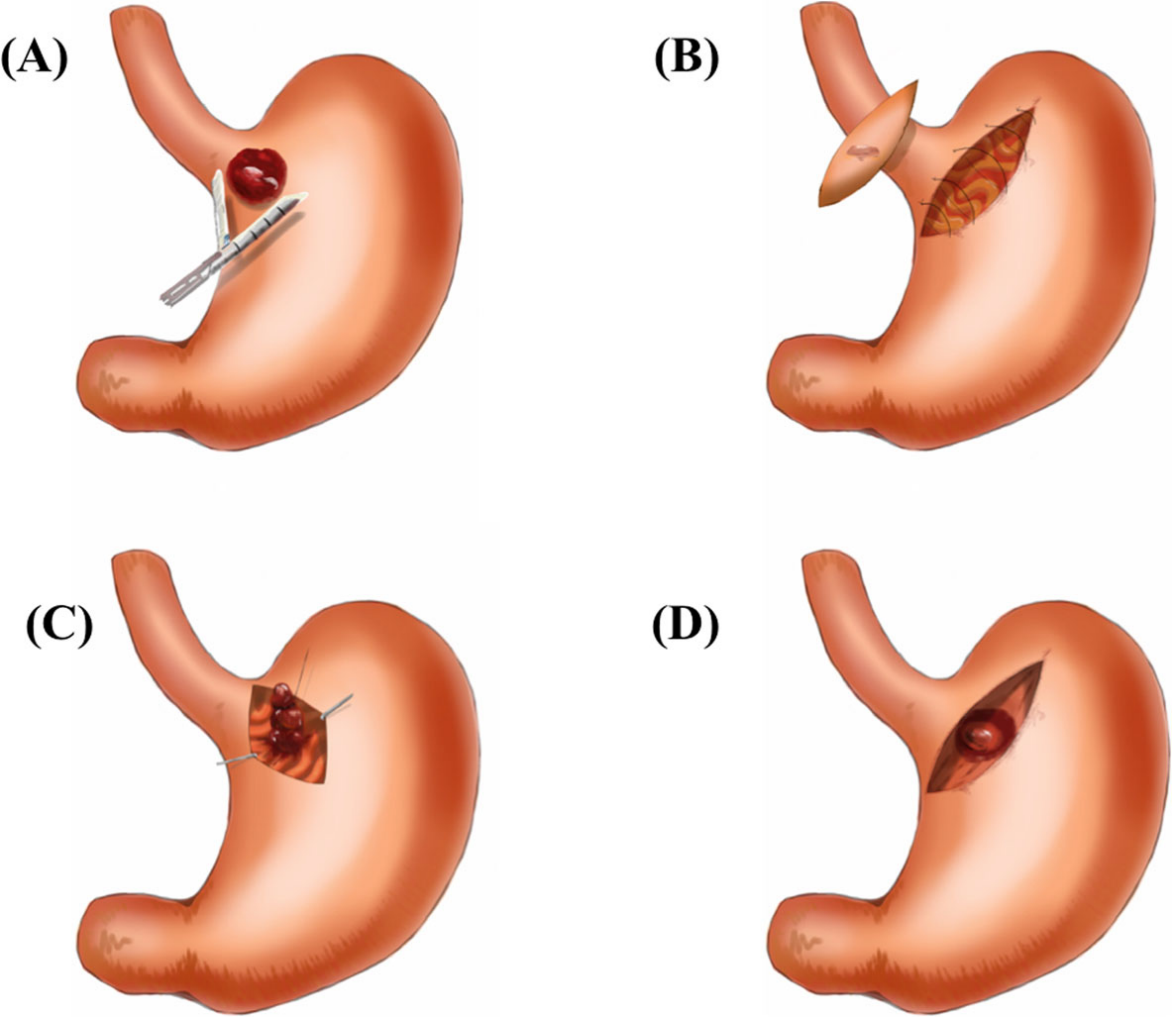
The different approaches of minor gastrectomy with MIS. **a** Resection with surgical staplers. **b** Whole-layer resection and repair. **c** Gastrotomy then intra-gastric resection. **d** Serosal approach for resection

The cohort (119 patients) was stratified into three groups according to the extent of gastrectomy. Thirteen patients underwent either proximal gastrectomy or total gastrectomy (all by laparotomy) and they were designated as the major gastrectomy group. For the remaining 106 patients who underwent gastric wedge resection (minor gastrectomy), 51 of them (48%) underwent laparotomy, whereas the other 55 patients (52%) underwent surgery with the laparoscopic approach (Fig. 2). None of the patients in the laparoscopic group were converted to laparotomy. The methods of partial gastrectomy in the laparoscopic group included direct resection with staplers (*n* = 46), whole-layer resection with intracorporeal repair by sutures (*n* = 5), gastrotomy for tumor resection (*n* = 3), and resection with a serosa side approach without luminal penetration (*n* = 1) (Fig. 3). The decision to perform serosa side approach resection was based on the CT scan, which disclosed a more prominently protruding tumor at the lesser curvature side of EGJ other than the mucosa side. There was only one SMT presented with this picture in our cohort. On the other hand, for tumors locating at the posterior wall without remarkable serosa protrusion, gastrotomy from the anterior wall followed by whole layer resection of the stomach was applied. The clinicopathologic features of the three groups are summarized in Table 1.

**Table 1:** The clinicopathological features of the cohort

	(a) Major gatrectomy	(b) Minor gastrectomy by laparotomy	(c) Minor gastrectomy with MIS			
				*P*-value (a)(c)	(b)(c)	(a)(c)
Number	13	51	55			
Age (year)	50 ± 17	60 ± 15	61 ± 14	0.089	0.897	0.044
Gender						
M	8	27	24			
F	5	24	31			
Tumor size (cm)	5.3 ± 1.9	4.1 ± 1.8	3.7 ± 1.4	0.041	0.457	0.005
Pathology						
GIST	7	35	47			
Leiomyoma	6	15	5			
Schwannoma	0	1	3			
Others	0	0	0			
OP duration (min)	205 ± 80	138 ± 53	126 ± 49	< 0.001	0.486	< 0.001
Hospital stay (day)	12 ± 3.6	10 ± 3.1	7 ± 2.4	0.097	< 0.001	< 0.001
Complications	3	6	1			
Margin *	2	1	1			
Recurrence *	2		1			
Follow-up * (months)	72.9		40.4			

*MIS* minimally invasive surgery, *GIST* gastrointestinal stromal tumor;

* remark the parameters specified for GISTs

**Fig. 4:**
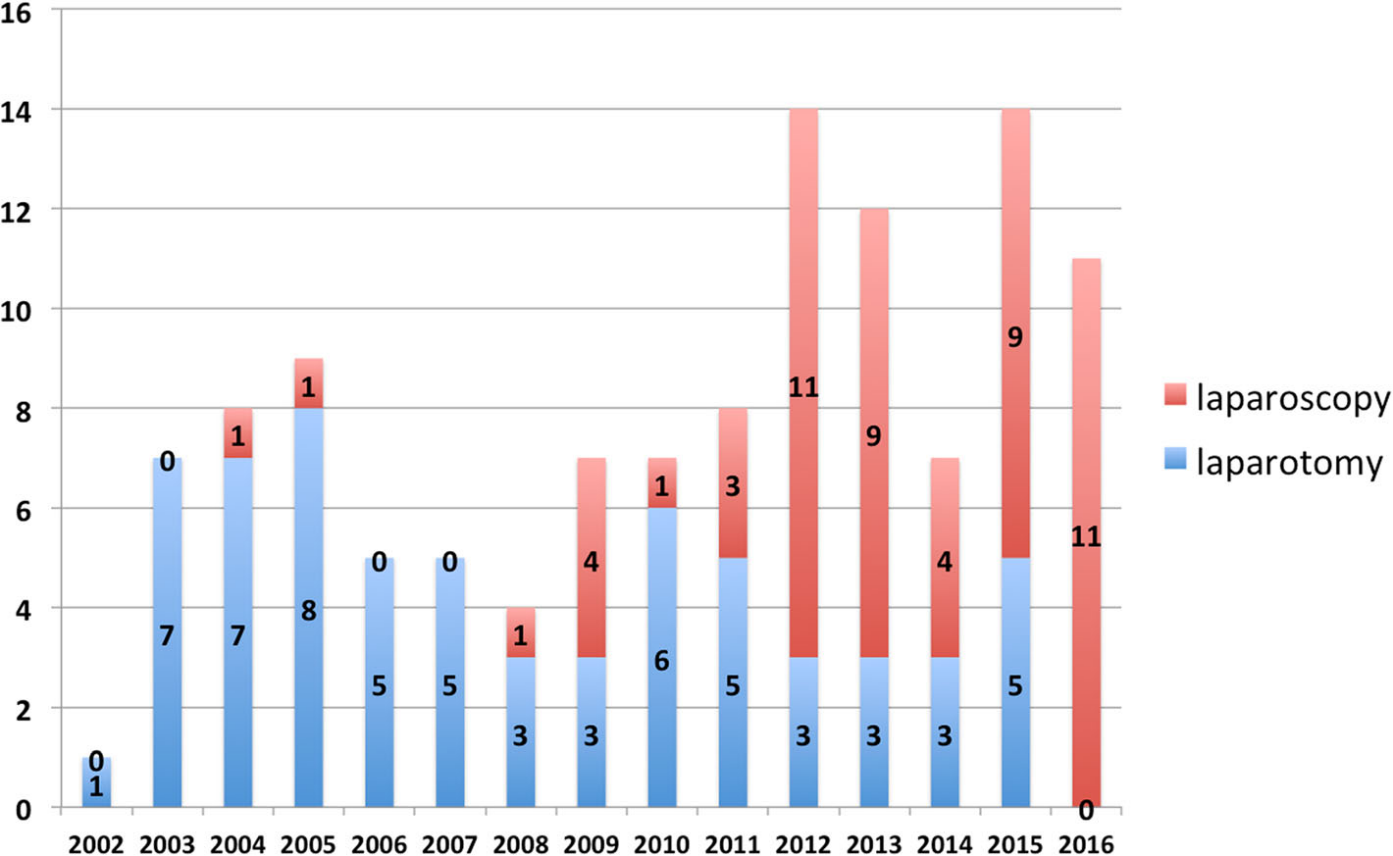
The annual numbers of SMTs around the EGJ underwent resection by the two approaches

The average tumor size was significantly larger in the major gastrectomy group than in the minor gastrectomy groups (5.33 cm, 4.07 cm, and 3.69 cm, respectively; *p* = 0.041 and 0.005, respectively). However, there was no significant difference in the average tumor size between the two minor gastrectomy groups (laparotomy versus laparoscopic approach). Similar results were also observed in the other associated parameters: the minor gastrectomy group that underwent laparoscopic surgery had the shortest operation duration and hospital stay among the three groups. Figure 4 demonstrates the annual number and distribution of surgical approaches for patients who underwent minor gastrectomy. More MISs have been applied than conventional laparotomy since 2012. In 2016, all of the SMTs around the EGJ were resected laparoscopically.

Based on the endoscopic view from the stomach to the EGJ, we divided the defined area of the EGJ and the 106 SMTs underwent minor gastrectomy into 4 zones (Fig. 1): zone 1 included tumors located at the ventral aspect (anterior wall) of the EGJ; zone 2 contained tumors located at the fundus (greater curvature) side of the EGJ; zone 3 included tumors located at the dorsal side (posterior wall) of the EGJ; and zone 4 contained tumors located at the lesser curvature side of the EGJ area. The majority of tumors located in zone 2 were resected laparoscopically (82% versus 18%, *p* = 0.001). For SMTs in the zone 1, 3, and 4, they were more often resected by laparotomy although there was no significant difference between each approach (Table 2). Excluding SMTs in the zone 2, the percentage of resection performed by laparotomy was 59%.

**Table 2:** The number of SMTs around EGJ underwent minor gastrectomy by laparotomy and with MIS

	Minor gastectomy by laparotomy		Minor gastrectomy With MIS		*P*-value
Resection method	By stapler	Without stapler	By stapler	Without stapler	
Zone 1	0	7 (70%)	2 (20%)	1 (10%)	0.206
Zone 2	2 (7%)	3 (11%)	21 (75%)	2 (7%)	0.001
Zone 3	2 (6%)	17 (48%)	14 (40%)	2 (6%)	0.612
Zone 4	0	20 (61%)	9 (27%)	4 (12%)	0.223
Total	4	47	46	9	

*MIS* minimally invasive surgery

In the major gastrectomy group, there were 3 complications among 13 patients (2 intra-abdominal abscesses adjacent the anastomosis and 1 laparotomy wound infection, 3/13 = 23.1%). Both of the intra-abdominal abscesses were managed with percutaneous CT-guide drainage and parenteral antibiotic administration. These two patients were discharged on postoperative day 17 and day 22, respectively. The patient suffered from wound infection was managed with wound care at ward and discharged on postoperative day 15. There were 6 complications in the laparotomy minor gastrectomy group (5 leakages with abscess and 1 case of postoperative bleeding, 6/51 = 11.8%), while there was only 1 complication in the laparoscopic minor gastrectomy group (postoperative pneumothorax, 1/55 = 1.8%). All of the leakages were confirmed by esophagography with water-soluble contrast, and CT scan of abdomen proved the existence of abscesses. One of the patients underwent endoscopic-guide nasojejunal tube placement for enteral feeding owing to delayed healing of leakage, while the remaining four patients were treated by parenteral nutrition support for their self-limited leakages.

Regarding the final pathology of the SMTs, there were 89 GISTs, 26 leiomyomas, and 4 schwannomas. Margin status is an important issue for the resection of GISTs. Among the 48 GISTs resected by laparotomy (including major and minor gastrectomy), 3 patients achieved R1 resection unfortunately, whereas there was one tumor with a positive margin among the rest of the GISTs that were resected laparoscopically. Furthermore, 2 patients suffered from recurrent GISTs in the former cohort, and one patient suffered from a recurrent GIST in the latter cohort (the mean follow-up periods were 72.9 months and 40.9 months, respectively).

## Discussions

SMTs exist along the whole gastrointestinal tract from the esophagus to the rectum, although there is a specific prevalence among sites [12]. The presence of symptoms is one of the indications for upfront resection even if a preoperative pathologic diagnosis is not obtainable [13]. MISs have been proven to be safe and efficient in the resection of gastric and small bowel SMTs [14, 15]. For SMTs located in difficult locations, such as the esophagus, prepyloric area of the stomach, duodenum, and rectum, there is no consensus yet whether MIS is an approach superior to laparotomy. In addition to minimizing surgical morbidity and mortality, functional preservation is also a critical factor during surgery [16]. Our retrospective review revealed that patients with EGJ SMTs who underwent major gastrectomy had significantly larger tumors than those who underwent minor gastrectomy. In fact, most of the resections were performed before the concept of neoadjuvant targeted therapy [17, 18]. For GISTs located in difficult locations or with relatively large sizes, preoperative biopsy should be conducted to determine if the administration of neoadjuvant tyrosine kinase inhibitors (TKIs) can reduce the tumor size so that equal surgical and oncological results can be achieved by minimal organ resection while maintaining maximal functional preservation. Retrospectively speaking, seven patients in the major gastrectomy group whose SMTs were proven to be GISTs after the operation might benefit from the effects of perioperative TKIs and could undergo minor gastrectomy instead.

The surgical stapler system has the advantages of not requiring intracorporeal sutures. Minimally invasive GI surgery has rapidly developed since the implementation of the surgical stapler system to perform resection and anastomosis, and our cohort was no exception (Fig. 3). Our surgeons are willing to try MIS on tumors at difficult locations as they became more experienced and skillful. Except for the evolution of the approach over time, the review of our cohort demonstrated that the locations of SMTs is another critical factor to determine the approach of resection. SMTs on the lesser curvature side of the EGJ (zone 4) were more often resected with conventional laparotomy, whereas the majority of SMTs on the greater curvature side (zone 2) were resected laparoscopically (Table 2). In the MIS minor gastrectomy group, 46 of them (84%) were resected with surgical staplers, which did not require additional intracorpreal management. Regarding both the linear configuration of surgical staplers and the particular shape of the stomach, gastrectomy with surgical staplers on the lesser curvature side of the stomach is not as straightforward as that on the greater curvature side. This factor might have hindered our surgeons from performing MIS around the lesser curvature side of the stomach. This is even noteworthy for tumors around the EGJ. Considering the similar tumor sizes between the two minor gastrectomy groups demonstrated in our review, MIS should be attempted first and can result fewer complications and recovery days. The majority of SMTs located in the zone 3 and zone 4 were resected by conventional whole-layer wedge resection (Table 2), but an alternative method of resection should be adopted when surgical staplers cannot be applied straightforwardly. Laparoscopic endoscopic cooperative surgery (LECS) was first proposed by Hiki et al [19] as a hybrid approach for resection of GI SMTs. The SMT was identified from the mucosal side by endoscopy, and then submucosal dissection was performed. Subsequently, the surgeon performed seromuscular dissection from the peritoneal cavity along the landmarks made by endoscopic dissection to complete the resection. This hybrid method could achieve R0 resection with maximal preservation of the normal tissue, and our patients who underwent resection by laparotomy might potentially be rendered to MIS resection and benefit from this method [20].

The prominent side of the SMT is also a factor to determine the surgical approach. According to EGD and CT of each patient in the minor gastrectomy group, only one patient had a SMT more protruding at the serosa side, while the remaining patients had SMT only visible from the mucosa side. The SMT was resected from the serosa side successfully according to the preoperative planning (Fig. 3). The concept was inspired by LECS. Tumor rupture and spillage is a risk factor for recurrence after resection of GIST [21]. Regarding the 3 patients underwent gastrotomy followed by resection of tumor locating at the posterior wall, contamination of the aseptic peritoneal cavity is another potential risk of infection. A better approach for resection is warranted in the future.

Whether the resection is performed by laparotomy or with MIS, complete resection of the tumor with minimal complications is the first priority. For patients with GISTs, margin status is an additional issue. The conventional minor gastrectomy group included1 patient with a positive resection margin among 35 patients, whereas the minor gastrectomy group who underwent MIS included 1 patient with positive margins among 46 patients. Since there was no significant difference in tumor size between the two groups (same extent of gastrectomy), minor gastrectomy with laparoscopic surgery did not yield inferior oncologic results to conventional laparotomy.

There are some limitations regarding this study. First, the innate bias is the preference of surgeons since there are 7 surgeons performing MIS in our division. Each of them are experienced and with varied confidence in performing MIS for SMTs around EGJ. Second, the exact distance between the tumor and the EGJ might be different based on the pressure of insufflation during EGD. Third, the tumor size was represented by the longest axis, which was different on the preoperative EGD/ CT scan, and by the postoperative pathologic examination. Nevertheless, this retrospective review showed that MIS minor gastrectomy was eligible with equal safety and oncologic result. Based on this observation, a prospective study in the management of SMTs around EGJ is warranted to establish the paradigm of management in the future.

## Conclusions

For SMTs around the EGJ area, larger tumors (diameter of more than 5 cm) are more likely to be resected with major gastrectomy. On the contrary, smaller tumors were resected with non-anatomical gastrectomy. Furthermore, to resect SMTs around the EGJ in a wedge-like (minor gastrectomy) fashion, tumors located on the greater curvature side were more often resected with MIS, unlike those in the other quadrants. Our study also demonstrated that MIS yielded acceptable safety and surgical outcomes compared to conventional laparotomy for SMTs around the EGJ of the same size.

## Data Availability

The dataset used and/or analyzed during the current study are available from the corresponding author on reasonable request.

## Abbreviations

SMT: submucosal tumor
GIST: gastrointestinal stromal tumor
CT: computed tomography
MIS: minimally invasive surgery
EGJ: esophagogastric junction
EGD: esophagogastroduodenoscopy
TKI: tyrosine kinase inhibitor
LECS: laparoscopic endoscopic cooperative surgery
